# Global O-GlcNAc Levels Modulate Transcription of the Adipocyte Secretome during Chronic Insulin Resistance

**DOI:** 10.3389/fendo.2014.00223

**Published:** 2015-01-22

**Authors:** Edith E. Wollaston-Hayden, Ruth B. S. Harris, Bingqiang Liu, Robert Bridger, Ying Xu, Lance Wells

**Affiliations:** ^1^Complex Carbohydrate Research Center, University of Georgia, Athens, GA, USA; ^2^Department of Biochemistry and Molecular Biology, University of Georgia, Athens, GA, USA; ^3^Department of Physiology, Georgia Health Sciences University, Augusta, GA, USA

**Keywords:** O-GlcNAc, insulin resistance, adipose tissue, adipocytokines, transcription

## Abstract

Increased flux through the hexosamine biosynthetic pathway and the corresponding increase in intracellular glycosylation of proteins via O-linked β-*N*-acetylglucosamine (O-GlcNAc) is sufficient to induce insulin resistance (IR) in multiple systems. Previously, our group used shotgun proteomics to identify multiple rodent adipocytokines and secreted proteins whose levels are modulated upon the induction of IR by indirectly and directly modulating O-GlcNAc levels. We have validated the relative levels of several of these factors using immunoblotting. Since adipocytokines levels are regulated primarily at the level of transcription and O-GlcNAc alters the function of many transcription factors, we hypothesized that elevated O-GlcNAc levels on key transcription factors are modulating secreted protein expression. Here, we show that upon the elevation of O-GlcNAc levels and the induction of IR in mature 3T3-F442a adipocytes, the transcript levels of multiple secreted proteins reflect the modulation observed at the protein level. We validate the transcript levels in male mouse models of diabetes. Using inguinal fat pads from the severely IR *db/db* mouse model and the mildly IR diet-induced mouse model, we have confirmed that the secreted proteins regulated by O-GlcNAc modulation in cell culture are likewise modulated in the whole animal upon a shift to IR. By comparing the promoters of similarly regulated genes, we determine that Sp1 is a common *cis*-acting element. Furthermore, we show that the LPL and SPARC promoters are enriched for Sp1 and O-GlcNAc modified proteins during insulin resistance in adipocytes. Thus, the O-GlcNAc modification of proteins bound to promoters, including Sp1, is linked to adipocytokine transcription during insulin resistance.

## Introduction

It is estimated that diabetes affects 8.3% of the United States population (1). Type 2 diabetes mellitus (T2DM) is characterized by both hyperinsulemia and hyperglycemia, which result from a combination of whole-body insulin resistance and pancreatic beta-cell dysfunction that leads to insulin insufficiency (2). T2DM can lead to a wide-range of severe and costly complications, such as blindness, kidney failure, stroke, and cardiovascular disease (3). The abundance of associated complications reflects the number of interrelated systems involved in T2DM pathogenesis (4).

White adipose tissue is an important mediator of energy homeostasis. In addition to its role as an energy storage depot, it acts as an endocrine organ by secreting adipocytokines, such as leptin and adiponectin. Adipocytokines can affect both local and distant tissue insulin sensitivity and energy homeostasis (5, 6). Obesity alters the ability of adipose tissue to properly express and secrete adipocytokines. Obesity, which affects more than 10% of adults world-wide, is the leading environmental risk factor for the development insulin resistance and T2DM (7–9). Importantly, several adipocytokines have been implicated in the development of insulin resistance and the pathogenesis of T2DM (10). The mechanism by which adipocytes respond to the excess nutrient-flux during obesity and insulin resistance and alter the secretion of adipocytokines is not completely understood.

One way for cells to sense nutrient abundance and thereby alter their metabolism and gene expression is through the hexosamine biosynthetic pathway (HBP). In 1991, Marshall et al. first implicated the HBP in the development of insulin resistance (11). The HBP has been proposed to be a nutrient-flux sensor, since it utilizes 2–5% of intracellular glucose, and acts to limit the amount of glucose uptake by inducing insulin resistance (11). The end product of the HBP, uridine 5′-diphospho-*N*-acetylglucosamine (UDP-GlcNAc), is the sugar donor for the enzyme O-GlcNAc transferase (OGT), which transfers the O-GlcNAc post-translational modification onto serine or threonine residues of nuclear and cytoplasmic proteins (12–15). It has been demonstrated by many groups, including our own, that in multiple systems the elevation of O-GlcNAc levels is sufficient to induce insulin resistance (16–21).

The expression of several adipocytokines has been shown to be regulated at the level of transcription (22–26). Additionally, the transcription and secretion of several adipocytokines is modulated by altered HBP flux (22, 27, 28). Transgenic mice overexpressing OGT in peripheral tissues have both glucose disposal defects and hyperleptinemia, suggesting that the O-GlcNAc modification is intricately tied to the development of insulin resistance and the regulation of adipocytokines (16).

We have recently used shotgun proteomics to identify multiple murine secreted proteins from adipocytes (adipocytokines) whose levels are modulated upon the induction of insulin resistance by indirectly and directly modulating O-GlcNAc levels (29). In this study, we investigate the transcriptional regulation of several of the secreted proteins identified by proteomics. We explore whether O-GlcNAc modified transcription factors are regulating these proteins, since several adipocytokines are known to be regulated at the level of transcription and O-GlcNAc has been demonstrated to modify and alter the function of many transcription factors (30, 31). Here, we show that these secreted factors are co-regulated in a mouse adipocyte cell line and two mouse models of insulin resistance. We demonstrate that the promoters of these genes contain a common *cis*-acting motif for Sp1. We determine that Sp1 is more heavily O-GlcNAc modified during insulin resistance. Finally, we determine that Sp1 and O-GlcNAc modified proteins are enriched on the LPL and SPARC promoters. Our findings suggest that the O-GlcNAc modification of proteins regulates adipocytokine transcription during chronic insulin resistance.

## Materials and Methods

### Materials and reagents

Tissue culture media, serum, and antibiotics were purchased from Gibco (Grand Island, NY, USA). 3-isobutyl-1-methyxanthine and dexamethasone were from Sigma (St. Louis, MO, USA). Recombinant insulin, human, was from Roche Diagnostics (Indianapolis, IN, USA). O-(2-acetamido-2-deoxy-d-glucopyranosylidene)amino *N*-phenyl carbamate (PUGNAc) was from Toronto Research Chemicals Inc. (North York, ON, USA). GlcNAcstatin was a kind gift from Dr. Daan van Aalten (University of Dundee, Dundee, Scotland). Anti-Sp1 (PEP 2), anti-LPL (H-53), anti-Angiotensin I/II (N-10), anti-ERK-2 (C-14), normal sera, and agarose conjugated beads were from Santa Cruz Biotechnology (Santa Cruz, CA, USA). Anti-PEBP1 was from Novus Biologicals (Littleton, CO, USA). Anti-SPARC was from Abcam (Cambridge, MA, USA). Anti-O-GlcNAc (RL2) was from Enzo Life Sciences (Farmingdale, NY, USA). Anti-O-GlcNAC (CTD110.6) was previously generated in Dr. Gerald W. Hart’s Laboratory (Johns Hopkins University, Baltimore, MD, USA). Dynabead Protein G was from Life Technologies (Carlsbad, CA, USA).

### Cell culture and treatments

3T3-F442a preadipocytes were maintained and differentiated as previously described (29, 32). On day 6 after the induction of differentiation, the adipocytes were maintained in the appropriate low (1.0 g/L) or high glucose (4.5g/L) DMEM media containing 10% FBS, antibiotics, and vitamins with or without 100 μM PUGNAc, 20 nM GlcNAcstatin, or 100 nM insulin. After 24 h incubation, cells were washed either three times or five times (for media immunoblotting) with low or high glucose serum free media without antibiotics and vitamins. Following the rinses, cells were incubated for 16 h in the appropriate low or high glucose media without serum, antibiotics, and vitamins and with or without 100 μM PUGNAc, 20 nM GlcNAcstatin, or 1 nM insulin. After the incubation, the conditioned media was carefully collected, filtered, and buffer exchanged as previously described (29). The remaining cells were washed two times with ice cold PBS and then harvested by scraping and stored at −80°C until further analysis.

### Animals

Animal procedures were approved by the Institutional Animal Care and Use Committee of the University of Georgia. Animals were group housed with a 12-h light, 12-h dark cycle. Inguinal and retroperitoneal fat tissues from 12-week-old-male C57BL/6J wt (wt), C57BL/6J *db/db* (6J), and C57BL/3J *db/db* (3J) mice were isolated along with serum. Mice were fed *ad libitum* normal rodent chow. After sacrifice by decapitation, the inguinal fat was weighed, snap frozen, and stored at −80°C until transcript analysis. Both the 3J and 6J mice had both inguinal and retroperitoneal fat masses three times greater than the wildtype littermates as well as elevated serum glucose levels (>1.5×) and insulin levels (>5×) with the 3J mice having higher levels than 6J mice. For the diet-induced insulin resistance experiment, young (~9 weeks) C57BL/6 male mice were purchased from The Jackson Laboratory (Bar Harbor, ME, USA). Both treatment groups were fed *ad libitum* normal rodent chow and water. The mice in the high fat high sucrose (HFHS) treatment group were given free access to a 30% sucrose solution and lard in addition to their normal chow and water. After week 1, 2, and 3 of treatment, an insulin sensitivity test (ITT) was performed on a pair of mice closest to the average weight of each treatment group. The insulin sensitivity test was performed as previously described (33). Weights were recorded every week. After 3 weeks of treatment, six mice from each treatment group were sacrificed by decapitation. Trunk blood was collected for the measurement of serum insulin using a LINCO rat insulin RIA kit (EMD Millipore Corporation, Billerica, MA, USA). The liver and four fat pads (inguinal, epididymal, mesenteric, and retroperitoneal) were weighed, snap frozen, and stored at −80°C until transcript analysis.

### Cell lysates, Western blotting, and immunoprecipitation

For immunoprecipitations and anti-O-GlcNAc Western blots, 3T3-F442a cell pellets were lysed in 20 mM Tris pH 7.5, 150 mM NaCl, 1 mM EDTA, 1% NP-40, 1:100 protease inhibitor cocktail set V, EDTA-free (Calbiochem), and 1 μM PUGNAc. Protein concentration was determined using the Pierce BCA Protein Assay Kit (Thermo Scientific, Rockford, IL, USA). The CTD110.6 Western blots were performed essentially as described (34). Immunoprecipitations were carried out at 4°C overnight using anti-Sp1 or normal rabbit IgG with 750 μg of precleared protein lysate. Immunocomplexes were collected using Protein A/G-PLUS agarose beads for 2 h. Beads were washed four times with a modified RIPA buffer (20 mM Tris pH 7.5, 150 mM NaCl, 1 mM EDTA, 1% NP-40, 0.1% SDS) and one time with a high salt modified RIPA buffer (same as above except 500 mM NaCl). Proteins were eluted by boiling beads in 1× laemmli buffer and then transferred to a fresh tube for Western blotting. For the concentrated media Western blots, the protein concentration of the concentrated 3T3-F442a media was determined using the Bradford method and verified by Coomassie staining. Equal amounts of protein were separated by SDS-PAGE with Tris–HCl precast minigels (Bio-Rad, Hercules, CA, USA) and transferred to poly(vinylidene difluoride) (PVDF) membranes (for concentrated media) or nitrocellulose membranes (for immunoprecipitations) for Western blot analysis. After blocking for at least 1 h, membranes were incubated with the appropriate primary antibody overnight at 4°C. Membranes were incubated with the appropriate horseradish peroxidase-coupled secondary antibodies for 1 h, followed by extensive washing and Pierce ECL detection. ImageJ was used for densitometry (35).

### Chromatin immunoprecipitation

Chromatin Immunoprecipitations were performed as in the Millipore EZ-ChIP kit with some modifications. Day 8 adipocytes were washed once with room temperature PBS and then crosslinked by adding 1% formaldehyde in PBS and incubating for 10 min. The adipocytes were washed three times with cold PBS and then harvested by scraping. The adipocytes were resuspended in hypotonic lysis buffer (20 mM Tris–HCl, pH 7.5, 10 mM NaCl, 3 mM MgCl_2_, 1:100 Calbiochem protease inhibitor) and incubated on ice then dounce homogenized. The nuclei were collected by centrifugation and then resuspended in SDS lysis buffer. DNA was sheared to between 200 and 1000 base pair fragments using a Misonix S-4000 sonicator. Protein concentration was quantified using the Pierce BCA Protein Assay Kit. One hundred micrograms of chromatin was used per immunoprecipitation. Sonicated chromatin was diluted 1:10 with dilution buffer and precleared using Protein A/G-PLUS agarose, normal goat IgG, and sheared salmon sperm DNA (ssDNA) (Ambion). Three percent of the sample was saved as Input. One microgram of anti-Sp1, anti-O-GlcNAc (RL2), or normal IgG was used for the immunoprecipitation. Immunocomplexes were collected for 1 h using Protein G Dynabeads that were blocked with ssDNA and BSA (New England Biolabs). The Dynabeads were washed five times and then eluted with 1% SDS and 0.1 M NaHCO_3_. The elutions were decrosslinked at 65°C overnight with NaCl and RNase A (Ambion). After Proteinase K treatment (New England Biolabs), samples were purified by a Phenol–Chloroform extraction followed by ethanol precipitation overnight at −20°C using glycogen as a carrier. Precipitated DNA was resuspended in 3 mM Tris–HCl pH 8.0, 0.1 mM EDTA. qPCR was performed using primers for the proximal mouse SPARC and LPL promoter Sp1-binding sites. Sequences of primers were SPARC primers 5’-AGGCAAGTTCACTCGCTGGCT-3’ (forward) and 5’-AGACACCCTGGCCCCACCTG-3’ (reverse) and LPL primers 5’-CCTTCTTCTCGCTGGCACCGTT-3’ (forward) and 5’-GGGCAGAACAGTTACAAGGGGCA-3’ (reverse). The fold enrichment was calculated for each primer/antibody/treatment combination. First the normalized ChIP *C*_t_ values were calculated: Δ*C*_t(normalized ChIP)_ = {*C*_t(ChIP)_ − [*C*_t(Input)_ − Log_2_ (Input Dilution Factor)]}. The % Input was calculated: % Input = 2 ^(−ΔCt [normalized ChIP])^. Lastly, fold enrichment was calculated: fold Enrichment = (% Input of antibody/% Input of IgG).

### Gene expression analysis

RNA was isolated from 3T3-F442a cell pellets and inguinal fat pads using the Invitrogen PureLink Micro-to-Midi RNA Total RNA Purification System with Trizol reagent and on column DNase I treatment. The Invitrogen Superscript III First-Strand Synthesis System for RT-PCR was used to synthesize cDNA (Life Technologies, Carlsbad, CA, USA). All RT-qPCR primers were obtained from Qiagen QuantiTect Primer Assays and used with Qiagen QuantiTect SYBR Green PCR Kits (Qiagen, Valencia, CA, USA). ChIP-qPCR primers were used with iQ SYBR Green Supermix (Bio-Rad, Hercules, CA, USA). Amplifications were performed in a Bio-Rad 96-well iCycler or myIQ real-time detection system using the appropriate QuantiTect or iQ SYBR Green cycling protocol. Changes in target gene expression were normalized to TATA box binding protein (Tbp) and ribosomal protein L4 expression (Rpl4). Relative transcript levels were calculated using the ΔΔCt method (36). Normoglycemic transcript levels were set to 100.

### Motif analysis

Promoter sequences containing 500 bp upstream of the transcriptional start site were collected for human, mouse, and rat using the UCSC Genome Browser (37). No rat ortholog was found for Quiescin Q6. The human set was used as the main set and was supported by the mouse and rat ortholog sets. Three genes that were identified in the rodent adipocyte secretome but did not change in expression during insulin resistance were used as the negative set for human, mouse, and rat (29). Seven motif finding tools were used for primary motif finding: AlignACE (38), Bioprospecter (39), CONSENSUS(40), CUBIC (41), MDscan (42), MEME (43), and BOBRO (44). For each candidate, a position weight matrix and scoring matrix were generated (Table S1 in Supplementary Material). Corresponding transcription factor binding motifs were determined by analyzing the position weight matrix with TOMTOM (45). Conserved transcription factor binding motifs were confirmed using human and mouse sequences in rVISTA 2.0 (46).

### Statistical analysis

All statistics were performed using the General Linear Model Analysis of Variance [GLM AOV, Statistix (Statistix 10.0, 2010, Tallahassee, FL, USA)]. Error bars represent the SEM of independent experiments. *P*-values under 0.05 were considered significant and represented using an * in all figures. All experiments shown were replicated three to five times.

## Results

### The induction of insulin resistance in 3T3-F442a adipocytes modulates secreted steady-state protein levels and transcript levels in the same manner

**Figure 1:**
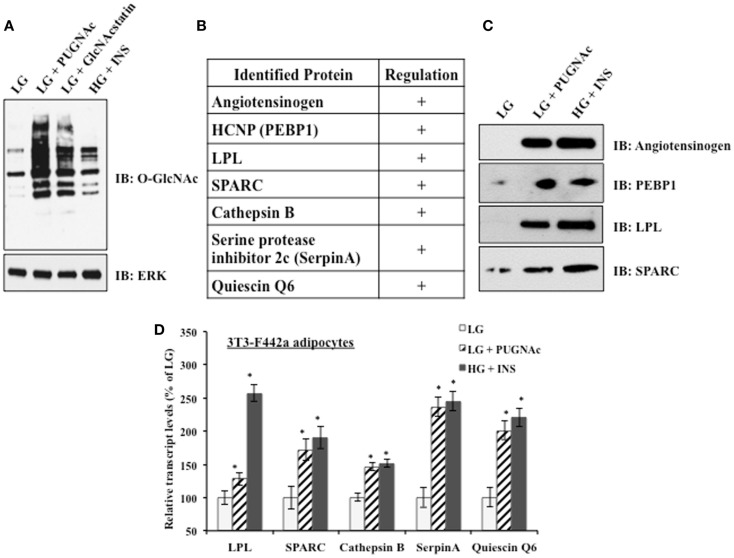
**Insulin resistant 3T3-F442a adipocytes display altered adipocytokine expression**. **(A)** 3T3-F442a adipocytes were grown under insulin responsive (LG) or insulin resistant (LG with PUGNAc, LG with GlcNAcstatin, or HG with insulin) conditions as described in Section “Materials and Methods.” Equal amounts of protein from whole cell lysates were separated by SDS-PAGE and Western blotting was performed using anti-O-GlcNAc (CTD110.6). Equal loading was confirmed by Western blotting with ERK2. **(B)** A partial list of rodent adipocytokines found to be regulated by insulin resistance based on proteomic quantification. **^+^**Indicates that the protein expression is upregulated upon the induction of insulin resistance. **(C)** Proteomic quantification of protein expression was confirmed by Western blotting in 3T3-F442a adipocytes. Equal amounts of concentrated media were separated by SDS-PAGE and Western blotting was performed with the designated antibodies. **(D)** The steady-state transcript levels were evaluated using qPCR in 3T3-F442a adipocytes. Data are presented so that 100% represents the transcript level in the insulin responsive condition (LG). *P < 0.05.

3T3-F442a preadipocytes were differentiated into mature adipocytes before experimental treatments. Mature adipocytes were either maintained in insulin sensitive conditions [low glucose (LG)] or shifted to insulin resistant conditions by the classical treatment of high glucose and chronic insulin (HG + INS) to generate hyperglycemia and hyperinsulemia or by treatment with low glucose and the OGA inhibitors PUGNAc (LG + PUGNAc) or GlcNAcstatin (LG + GlcNAcstatin) to more specifically elevate global O-GlcNAc levels. Figure 1A shows that all insulin resistant conditions generated elevated global O-GlcNAc levels as evaluated by immunoblotting with an O-GlcNAc specific antibody. Previously, our group used shotgun proteomics to characterize the secreted proteome of rodent adipocytes and to identify multiple proteins whose levels are modulated upon the induction of insulin resistance by indirectly and directly modulating O-GlcNAc levels in rodent adipocytes as described above (29). Figure 1B shows a shortened list of adipocytokines whose protein expression was found to be positively regulated by the induction of insulin resistance using quantitative proteomics in our previous studies. Here, we validated the relative levels of several of these secreted proteins using immunoblotting as an orthogonal method. 3T3-F442a adipocyte conditioned media from each treatment group was concentrated and buffer exchanged before immunoblotting with selected antibodies. Figure 1C shows that the regulation observed by quantitative proteomics is recapitulated by immunoblotting as an independent method. Since adipocyte insulin resistance was induced by either indirectly (HG + INS) or directly (LG + PUGNAc) altering O-GlcNAc levels, it is likely that O-GlcNAc is modulating the secretion of these adipocytokines. Since the secretion of many of the adipocytokines studied thus far is regulated at the level of transcription (22–26) and O-GlcNAc has been shown to modify and alter the function of many transcription factors (47), we hypothesized that the elevation of O-GlcNAc levels was regulating many of the identified adipocytokines at the level of transcription. Figure 1D shows that upon the elevation of O-GlcNAc levels and the induction of insulin resistance in 3T3-F442a adipocytes, the steady-state transcript levels of many of the identified secreted proteins, as measured by qPCR, reflect the modulation observed at the protein level.

### The induction of insulin resistance modulates secreted protein steady-state transcript levels in a genetic insulin resistant mouse model

**Figure 2:**
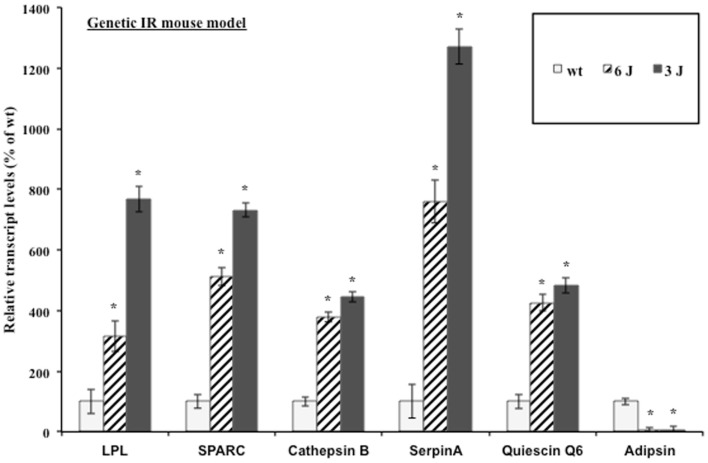
**Genetic insulin resistant mice display altered adipocytokine steady-state transcript levels**. Inguinal fat pads from 12-week-old male *wt*, *6J*, and *3J* (*n* = 6) mice were used for transcript analysis by qPCR. Data are presented so that 100% represents the transcript level in the insulin responsive condition (*wt*). *P < 0.03.

The mouse preadipocyte cell lines are a very useful system for studying adipocyte biology; however, their ability to secrete proteins at the high levels measured *in vivo* is impaired in many cases (48). Additionally, the complex paracrine interactions between adipocytes and the stromal-vascular cell fraction that comprises adipose tissue as well as the signaling between tissues in a whole animal are lost in adipocyte cell lines *in vitro* (49). Therefore, the regulation of adipocytokine transcript levels upon the induction of insulin resistance was examined in a biologically relevant mouse model. The inguinal fat pads from severely insulin resistant 12-week-old male leptin receptor mutant (*db/db*) mice were used for transcript analysis. *db/db* mice produce leptin but fail to respond to it. The C57BL/6J *db/db* (6J) mice produce only the short-form leptin receptors (Ob-Ra, Ob-Rc, Ob-Rd) and the circulating form leptin receptor (Ob-Re) but not the long signaling form of the receptor (Ob-Rb). The C57BL/3J *db/db* (3J) mice produce only the circulating form leptin receptor (Ob-Re) (50). Figure 2 shows the inguinal fat pad transcript levels in the *db/db* mouse models vary significantly from the *wt* mice and reflect the modulation shown at the transcript and protein levels in the 3T3-F442a adipocytes. All of the transcripts were elevated with the exception of the control gene, adipsin. Adipsin transcript levels have been shown to be downregulated in many models of rodent obesity (51).

### The induction of insulin resistance modulates steady-state transcript levels in a diet-induced IR mouse model

**Figure 3:**
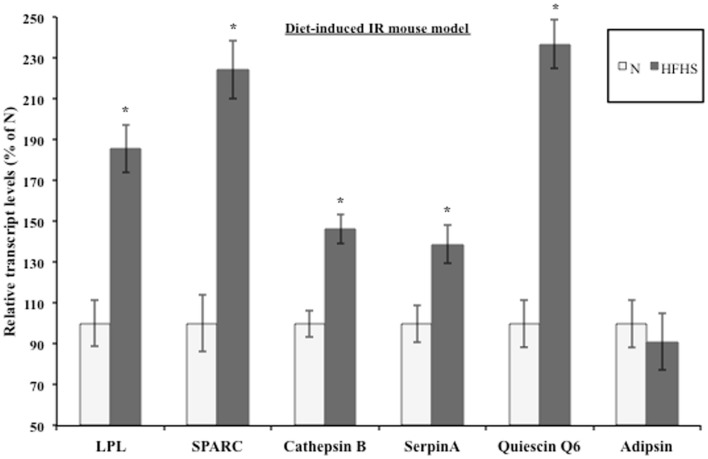
**Diet-induced insulin resistant mice display altered adipocytokine steady-state transcript levels**. Inguinal fat pads from C57BL/6 mice fed normal chow (N) (*n* = 4) or a high fat, high sucrose (HFHS) (*n* = 3) diet for 3 weeks were used for transcript analysis by qPCR. Data are presented so that 100% represents the transcript level in the insulin responsive condition (N). *P < 0.05.

Evidence suggests that T2DM develops from a combination of genetic and environmental factors but the relative contribution of each is unclear (52). A monogenic genetic mouse model (*db/db*) does not represent the true genetic heterogeneity that is present in most cases of human T2DM (53). In addition, the genetic defect is in an adipocytokine pathway, which could lead to potentially confounding effects for this experiment (54). To address these concerns, a diet-induced insulin resistant mouse model was developed by feeding *ad libitum* sucrose and lard (HFHS) to approximately 9-week-old C57BL/6 mice as described in Section “Materials and Methods.” After 3 weeks of treatment, the live weight as well as the wet weight of the inguinal, epididymal, mesenteric, and retroperitoneal fat pads was significantly increased in the HFHS mice compared to the mice on the normal chow diet (Figure S1A in Supplementary Material). The mice on the HFHS diet had elevated glucose levels and an attenuated response to insulin (Figure S1B in Supplementary Material). In addition, the mice displayed significantly elevated insulin levels (Figure S1C in Supplementary Material). After 3 weeks on the HFHS diet, the mice displayed mild insulin resistance and obesity so the inguinal fat pads were used for transcript analysis. Since the diet-induced insulin resistant mice were mildly obese and insulin resistant, we would expect the adipsin levels to only change slightly in contrast to the *db/db* mice, which were extremely obese and insulin resistant. Figure 3 shows the transcript levels were significantly elevated in the HFHS mice inguinal fat pads for all genes excluding adipsin. The diet-induced insulin resistant mice transcript levels reflect the modulation shown at the transcript level in the *db/db* mouse fat pads and at the transcript and protein levels in the 3T3-F442a adipocytes. Table 1 shows the relative increase in transcript levels in the insulin resistant mice (HFHS and 6J) compared to the transcript levels of the insulin sensitive mice (N and wt), which are set to 100%. Given that transcript level regulation is consistent for both insulin resistant mouse models, the transcript regulation observed in cell culture during insulin resistance is validated.

**Table 1:** **Secreted protein transcript levels elevated in mouse models of insulin resistance**.

Mouse model	LPL	SPARC	Cathepsin B	Serpin A	Quiescin Q6
Insulin sensitive	100	100	100	100	100
Diet-induced	185	224	146	139	237
Genetic	316	511	379	760	425

### Sp1 is a common cis-acting element for the adipocytokine promoters and the O-GlcNAc modification of Sp1 is altered during insulin resistance

**Figure 4:**
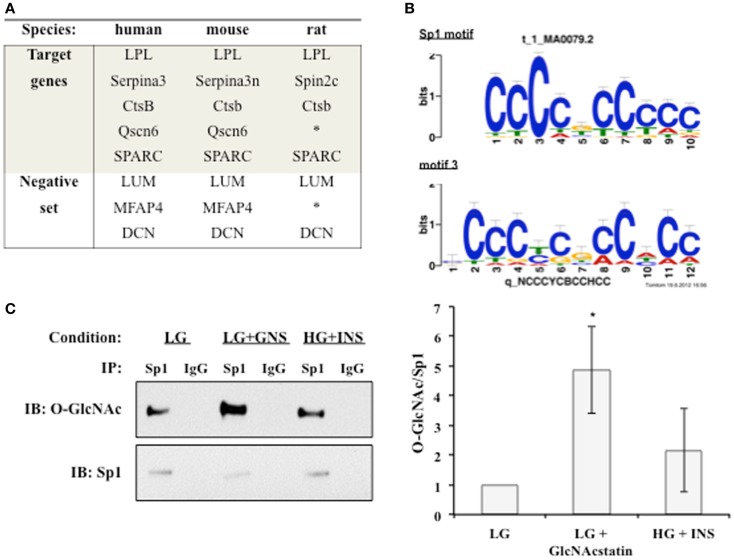
**An O-GlcNAc modified protein is identified as a common regulatory element**. **(A)** The promoters of target co-regulated genes for human, mouse, and rat were analyzed for common regulatory motifs as described in Section “Materials and Methods.” Three genes that were not co-regulated were used as a negative set to avoid identifying non-regulatory motifs. *Denotes no orthologous gene in rat. **(B)** TOMTOM was used to assign identified regulatory motifs to known transcription factor binding motifs. Regulatory motif 3 matches the Sp1 DNA binding motif with a *p*-value of 1.3 × 10^−6^. **(C)** Whole cell lysates from insulin responsive and insulin resistant 3T3-F442a adipocytes were subjected to immunoprecipitation with anti-Sp1 or normal rabbit IgG followed by immunoblotting with anti-Sp1 or anti-O-GlcNAc (RL2). A representative immunoblot is shown (*right panel*). The ratio of O-GlcNAc modified Sp1 to total Sp1 was quantified using densitometry of independent experiments (*N* = 4).

We hypothesized that a common transcription factor or cofactor was responding to the elevation of O-GlcNAc levels and altering the transcription of the observed secreted proteins. Multiple complementary motif finding programs were used to analyze the same set of orthologous proximal promoters in order to find a more accurate set of regulatory motifs. Human, mouse, and rat promoters were used to identify conserved motifs, with the hope that the most important regulatory motifs would be under stronger evolutionary pressure (Figure 4A) (55). Twenty-four common putative regulatory motifs were identified using motif analysis programs as described in Section “Materials and Methods” (Table S1 in Supplementary Material). The putative regulatory motifs were compared to known transcription factor binding motifs. The Sp1-binding motif was found to match putative regulatory motif 3 (Figure 4B). The conservation of the Sp1 sites between human and mouse promoters was verified using rVista 2.0. Sp1 is relevant to adipocytokine transcription since it is a target of the insulin signaling cascade and many promoters of genes regulated during insulin resistance have Sp1 motifs (56–61). In addition, Sp1 is known to be dynamically modified by O-GlcNAc (47). Sp1 O-glycosylation is reported to be elevated in the liver, kidney, and adipose tissue of *db/db* mice (62). Many studies have associated the altered O-GlcNAc modification of Sp1 with altered transcriptional activation of target genes (63–68). Figure 4C shows that immunoprecipitated Sp1 has greater O-GlcNAc modification during insulin resistance in 3T3-F442a adipocytes. Both direct (LG + GlcNAcstatin) and indirect (HG + INS) modulation of O-GlcNAc levels trended toward elevated Sp1 O-GlcNAc modification although only the GlcNAcstatin reached statistical signifcance. The more modest O-GlcNAc modification seen in the HG + INS condition was most likely due to the more modest increase in global O-GlcNAc levels (Figure 1A).

### Sp1 and O-GlcNAc modified proteins are enriched on the proximal SPARC and LPL promoters during insulin resistance

**Figure 5:**
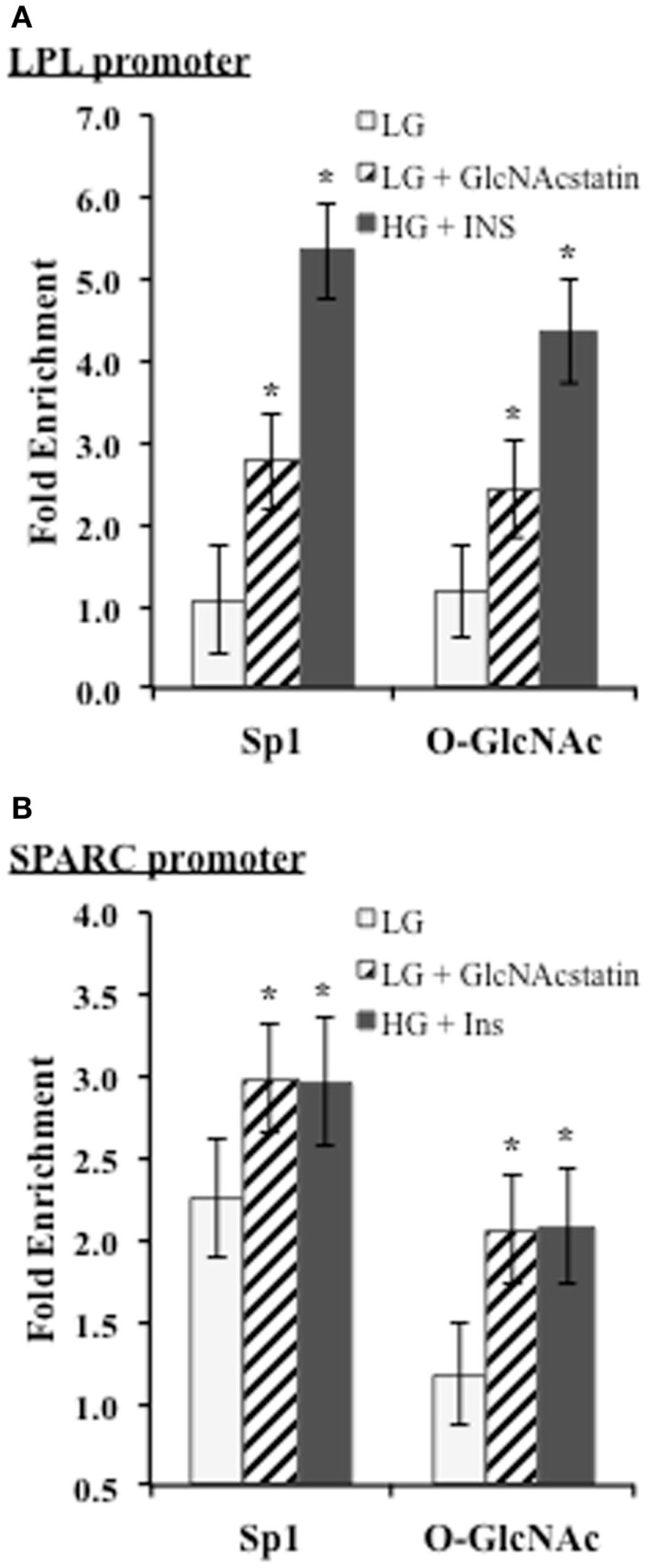
**ChIP analysis of conserved Sp1 sites on the SPARC and LPL promoters**. Insulin responsive and insulin resistant 3T3-F442a adipocytes were subjected to chromatin immunoprecipitation with anti-Sp1, anti-O-GlcNAc (RL2), or normal IgG. Quantitative PCR was performed with primers designed to amplify the conserved Sp1-binding site motif on the LPL **(A)** or SPARC **(B)** promoter. Fold enrichment was calculated using% Input as described in Section “Materials and Methods.” *P < 0.05.

We noticed that two of the identified motif three positions on the promoters corresponded with known biologically relevant Sp1-binding sites for LPL and SPARC. Since these sites are reported to be important for transcriptional activation, we wanted to determine whether Sp1 and O-GlcNAc modified proteins were enriched at these sites during insulin resistance in 3T3-F442a adipocytes. ChIP was performed with Sp1 and O-GlcNAc specific antibodies. Enrichment on the promoters was determined by analyzing purified DNA using qPCR with primers designed to amplify the region containing the Sp1-binding motif on either the LPL or SPARC promoter. Figure 5 shows both of the promoter regions showed significant enrichment of both Sp1 and O-GlcNAc modified proteins during insulin resistant conditions. These results suggest that the elevation of global O-GlcNAc levels, either directly or indirectly, leads to increased O-GlcNAc modification of Sp1 and increased Sp1 enrichment on the SPARC and LPL proximal promoters. Since the O-GlcNAc antibody will bind any protein modified with O-GlcNAc, the enrichment of O-GlcNAc on the LPL and SPARC promoters could be due to O-GlcNAc modified Sp1 or potentially other O-GlcNAc modified proteins.

## Discussion

White adipose tissue plays an important role in maintaining energy homeostatis by mediating lipid flux and altering the secretion of adipocytokines. Adipocytokines can act in an autocrine, paracrine, or endocrine manner to regulate a variety of processes, including energy homeostasis (5). Genetic mouse models showing that the induction of insulin resistance in white adipose tissue induces whole-body insulin resistance have highlighted the importance of adipocytokines during insulin resistance (69–71). In addition, adipocytokines are implicated in many of the complications leading to and resulting from T2DM, especially the tissue remodeling during nephropathy, cardiovascular disease, and obesity (72).

Many of the secreted proteins we studied are extracellular matrix (ECM) modulators and associated with inflammatory states. SPARC is a modulator of cell – ECM interactions and has diverse roles in osteogenesis, angiogenesis, fibrosis, tumorigenesis, and adipogenesis (73). Cathepsin B is associated with ECM degradation, apoptosis, and inflammation (74). SerpinA is an acute phase response protein that is involved in inflammation (75). Quiescin Q6 is upregulated in pancreatic cancer and may promote tumor cell invasion by upregulating matrix metalloproteinases (76, 77). Involvement in tumorigenesis is another common theme for these adipocytokines. During obesity, extensive remodeling is required for the expansion of fat pads (78). These ECM modulators may play an important role in local tissue remodeling. Obese adipose tissue is associated with an inflammatory response, which may also be mediated in part by these adipocytokines (78–83).

In this study, we have attempted to better define the relationship between O-GlcNAc modification and adipocyte-secreted protein transcription during insulin resistance. Several studies have suggested that leptin and adiponectin are regulated primarily at the level of transcription in adipocytes (22–25, 84). We investigated whether the secreted factors that we identified by quantitative proteomics were similarly regulated after confirming the elevation via an orthogonal method, Western blotting, for several of these secreted proteins. We have also recently evaluated many of these secreted factors in human adipose tissue with similar findings (85). We found that the induction of insulin resistance in mouse adipocytes elevated transcript levels in the same manner as protein levels for several of the secreted proteins identified by proteomics (Figure 1). Although a role for the transcriptional regulation of adipocytokine secretion has been established for SPARC (86), there are conflicting reports for LPL (87–90), and it was not known whether Cathepsin B, Quiescin Q6, and SerpinA were transcriptionally regulated in adipocytes. In addition, the biological relevance of the transcriptional upregulation of the proteins during insulin resistance was verified using both a genetic and diet-induced mouse model of insulin resistance (Figures 2 and 3).

Several studies have suggested that adipocytokine expression is regulated by the HBP and O-GlcNAc. Infusions of metabolites that increased HBP flux into rats increased leptin expression (22, 91). Both GFAT and OGT transgenic mice displayed hyperleptinemia (16, 27, 28). GFAT transgenic mice also displayed decreased adiponectin levels (28). In primary human adipocytes and 3T3-L1 mouse adipocytes, HBP flux was shown to correlate with leptin expression (27, 92). Although many studies have manipulated the HBP, studies that manipulate O-GlcNAc levels more directly and examine adipocytokine expression have been lacking. We found that both the direct modulation of O-GlcNAc levels by the addition of OGA inhibitors and the indirect modulation of O-GlcNAc levels by hyperglycemia and chronic hyperinsulinemia in mouse adipocytes elevated transcript levels in the same manner as protein levels (Figure 1). It was not known whether inducing insulin resistance solely by raising global O-GlcNAc levels would regulate these secreted proteins at the level of transcription.

It is reasonable to assume that co-regulated genes have a similar upstream regulator. Since we found that the expression of these proteins was similarly regulated by both classical insulin resistance and by solely raising global O-GlcNAc levels, we hypothesized that O-GlcNAc was a regulator. O-GlcNAc has been proposed to be a “nutrient sensor” because the levels of the end product of the HBP, UDP-GlcNAc, are regulated by the flux of glucose, uridine, glutamine, and FFA’s (93, 94). OGT is responsive to physiological levels of UDP-GlcNAc, so increased HBP flux results in globally elevated levels of O-GlcNAc modification (95). The regulation of OGT is complex and still being elucidated but it is clear that it has a preference for certain proteins and sites and does not universally add O-GlcNAc to all proteins (96–98). A large body of literature has shown that the O-GlcNAc modification plays an important role in transcriptional regulation. O-GlcNAc modifies transcription factors and cofactors, RNA Pol II, chromatin remodelers, and has even been identified as part of the histone code. O-GlcNAc modification of proteins can affect protein stability, protein–protein interactions, chromatin remodeling, transcriptional initiation and elongation, DNA binding, and localization (47, 99).

The secreted factors were regulated at the level of transcription, so we looked for common transcription factor binding motifs. After determining that Sp1 was a common *cis*-acting motif for these genes, we found that the O-GlcNAc modification of Sp1 trended toward an increased level during insulin resistance in mouse adipocytes (Figure 4). Sp1 has been implicated in the transcriptional regulation of LPL, SPARC, Cathepsin B, and SerpinA.

A role for Sp1 as a regulator of SPARC transcription has been established in transformed cells. The proximal promoter of SPARC contains several modified GC-boxes that are binding sites for Sp1 and/or Sp3. Sp1 and/or Sp3 are required for SPARC transcriptional activation in chickens, mice, and human beings (100–102). In chick embryonic fibroblasts, v-Jun represses SPARC promoter activation and initiates cell transformation by targeting the minimal promoter region. It was shown that v-Jun does not bind this DNA region directly but binds Sp1 and/or Sp3 to target promoter activation (101). c-Jun activates SPARC transcription in human MCF7 cells through the activation of Sp1 (100). In mammary carcinoma, Brg-1, a SWI/SNF chromatin remodeling complex ATPase, was shown to interact with Sp1 to activate SPARC transcription (102). Sp1’s involvement in SPARC transcription in adipocytes has not previously been described.

Several studies have associated Sp1 and/or Sp3 with LPL transcriptional regulation. Interferon-γ (IFNγ) decreases macrophage LPL transcription by decreasing Sp3 protein levels and Sp1 DNA binding to sites in the 5′ UTR, which is mediate by casein kinase 2 (CK2) and Akt (103, 104). Transforming growth factor-β (TGF-β) represses macrophage LPL transcription through Sp1 and/or Sp3 sites in the 5′-UTR (105). Sp1 and/or Sp3 also bind an evolutionarily conserved CT element (−91 to −83), also known as a GA box, in the proximal promoter. Sterols regulate LPL through a SRE site that is close to the CT element (89, 106). A T(−93)G SNP that is close to the CT element has been associated with a predisposition to obesity and familial combined hyperlipidemia in some studies in human beings. The minor allelic frequency is highly variable for difference ethnic populations and the SNP effect may be influenced by the synergistic effects of a Asp9Asn and T(-93)G haplotype that is present in some populations (107–110). People with both the Asp9Asn and T(-93)G mutations have been shown to have an increased risk of cardiac disease and decreased LPL activity in some studies (111–113). In the South African black population, the SNP was associated with mildly lower triglyceride levels and was associated with higher promoter activation in smooth muscle cells (108, 114). This is in contrast to other studies, which show that the mutation decreases Sp1 and/or Sp3 DNA binding leading to lowered transcriptional activation (106, 107, 109, 110). Sterol regulatory element-binding protein (SREBP) was found to act synergistically with Sp1 to activate the promoter in macrophages. Mutation of the CT element is also reported to decrease promoter reporter activity in 3T3-F442a pre-adipocytes (115). The importance of these Sp1/Sp3 binding sites has not been previously explored in mature adipocytes.

Both Sp1 and O-GlcNAc modified proteins were found to be significantly enriched in the region of the conserved Sp1 site on both the LPL and SPARC promoters (Figure 5). In our experiments in mature mouse adipocytes, Sp1 is most likely facilitating transcriptional activation. The studies described above have begun to shed light on the role of O-GlcNAc in modulating adipocytokine transcription through the modification of Sp1. Although Sp1 in ubiquitously expressed and often thought of as a housekeeping transcription factor, the diversity of Sp1 post-translational modifications and the wide-range of interaction partners can fine tune Sp1 activity in a context specific manner (116). Sp1 is subject to many forms of post-translational modification including phosphorylation, acetylation, sumoylation, ubiquitylation, and glycosylation. The sites of phosphorylation on Sp1 can either increase or decrease Sp1 DNA binding and transcriptional activation (117). Glycosylation can affect Sp1 stability, protein–protein interactions, DNA binding, degree of phosphorylation, and localization (47). These other modifications as well as the recruitment of other proteins to the promoters may explain the differences between HG + INS and LG + GNS. Sp1 has at least eight sites of O-GlcNAc modification, but the specific roles of each site is still being elucidated (118). Five sites of modification have been mapped to the DNA binding domain, and the mutation of these sites can disrupt Sp1 transcriptional activation in hepatocytes (62, 68). O-GlcNAc modification of the Sp1 activation domain inhibits Sp1 transactivation (64, 119). Since O-GlcNAc acts as a nutrient-flux sensor, many studies manipulate the glycosylation of Sp1 by manipulating nutrient flux. Studies using glycosylation site-specific Sp1 mutants would help to clarify the specific role of O-GlcNAc modification; however, determining the action of a specific glycosylation site could be complicated by the complex interplay between phosphorylation and O-GlcNAc modification as well as the presence of several other O-GlcNAc sites. In addition, site-specific Sp1 studies in adipocytes would be challenging since adipocytes are notoriously difficult to transfect. Other O-GlcNAc modified proteins could also be modulating the adipocytokine transcription since the O-GlcNAc enrichment on the promoters could be due to proteins other than Sp1. ChIP-reChIP would help to determine, which proteins are in complex on the promoters.

In conclusion, these experiments serve to identify a possible mechanism by which adipocytes respond to insulin resistance and regulate the expression of adipocytokines. Future work is aimed at identifying the specific function of the O-GlcNAc modification on Sp1 during insulin resistance in adipocytes. In addition, the mechanism of adipocytokine transcriptional upregulation in animal models should be investigated. Understanding the transcriptional regulation of adipocytokines by O-GlcNAc may provide therapeutic targets for normalizing the expression of adipocytokines during obesity and T2DM.

## Conflict of Interest Statement

The authors declare that the research was conducted in the absence of any commercial or financial relationships that could be construed as a potential conflict of interest.

## Supplementary Material

The Supplementary Material for this article can be found online at http://www.frontiersin.org/Journal/10.3389/fendo.2014.00223/abstract

Figure S1 **Characterization of the diet-induced IR mouse model. (A)** After 3 weeks on either the HFHS diet (*n* = 6) or normal diet (*n* = 6), mice were weighed, sacrificed, and the liver and four fat pads were dissected and weighed. Data are presented so that 100% represents the weight of the normal diet mice. **(B)** Insulin sensitivity test was performed as in Section “Materials and Methods.” **(C)** Trunk blood was used for an Insulin RIA. *P < 0.05.

Table S1 **Common motifs for adipocytokine promoters**.
