# Supplementary figures and images for: Global O-GlcNAc Levels Modulate Transcription of the Adipocyte Secretome during Chronic Insulin Resistance
Source: Front Endocrinol (Lausanne). 2015 Jan 22;5:223. doi: 10.3389/fendo.2014.00223 (PMC4302944; doi:10.3389/fendo.2014.00223)

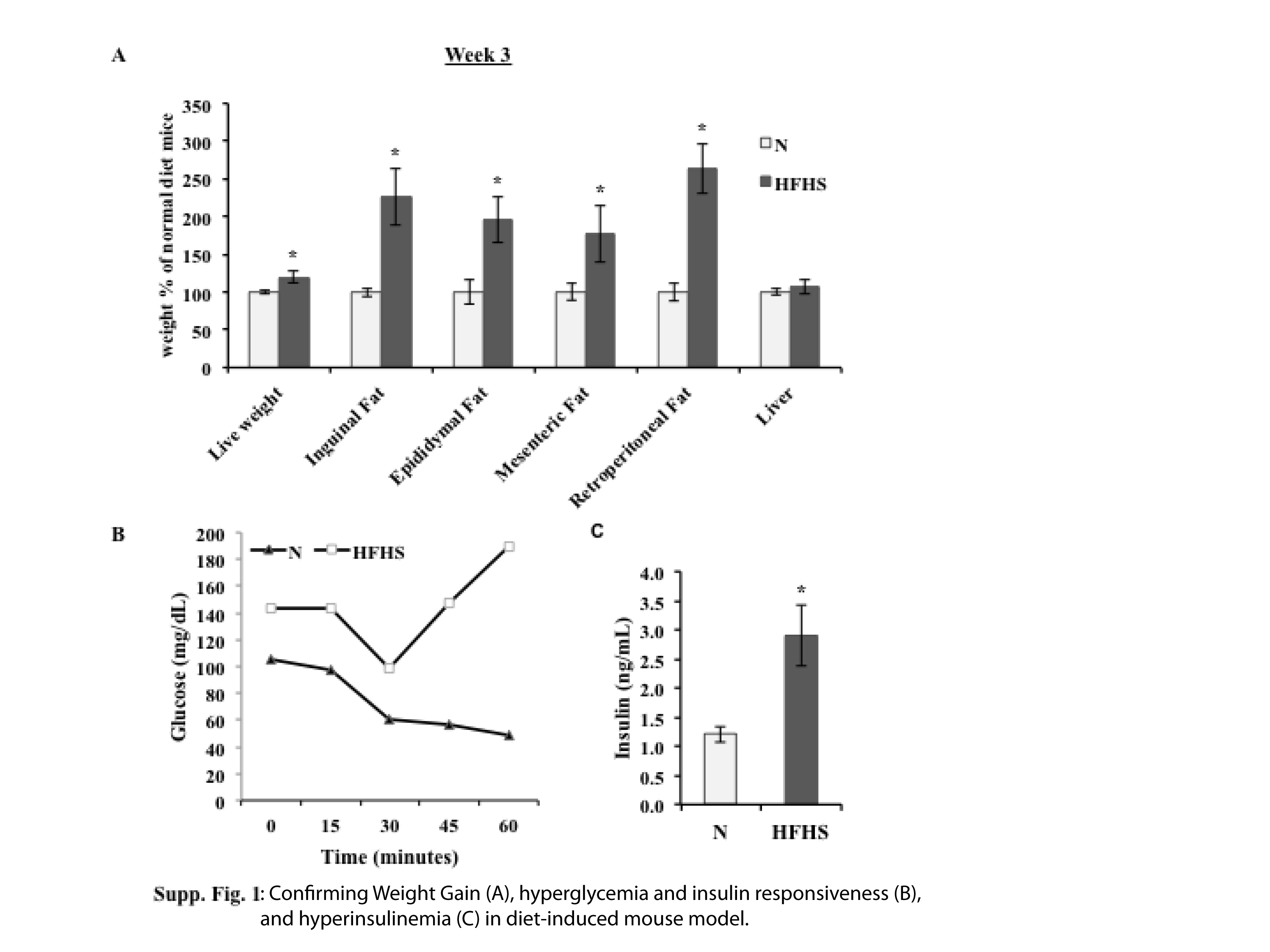

Supplement: Figure S1 — Characterization of the diet-induced IR mouse model. (A) After 3 weeks on either the HFHS diet (n = 6) or normal diet (n = 6), mice were weighed, sacrificed, and the liver and four fat pads were dissected and weighed. Data are presented so that 100% represents the weight of the normal diet mice. (B) Insulin sensitivity test was performed as in Section “Materials and Methods.” (C) Trunk blood was used for an Insulin RIA. *P < 0.05. [file Image_1.TIF]
