# Supplementary material for: Global O-GlcNAc Levels Modulate Transcription of the Adipocyte Secretome during Chronic Insulin Resistance
Source: Front Endocrinol (Lausanne). 2015 Jan 22;5:223. doi: 10.3389/fendo.2014.00223 (PMC4302944; doi:10.3389/fendo.2014.00223)
Supplement: Table S1 — Common motifs for adipocytokine promoters. [file Table_1.DOCX]

Supplementary Table S1. Common motifs for adipocytokine promoters

Motif Candidate 1

Motif length: 12; Motif number: 16; Consensus: AGAGGCCGGGAG

The profile matrix:

A 8 1 13 2 3 3 0 5 1 1 11 2

G 4 14 2 14 11 0 2 9 15 15 3 12

C 0 0 1 0 1 12 8 0 0 0 1 0

T 4 1 0 0 1 1 6 2 0 0 1 2

Motif Candidate 2

Motif length: 12; Motif number: 16; Consensus: CTGCCCTTTCCC

The profile matrix:

A 2 0 0 5 0 2 2 1 0 1 0 5

G 1 0 11 3 0 0 1 3 3 0 0 0

C 12 1 2 7 14 11 3 0 0 14 14 9

T 1 15 3 1 2 3 10 12 13 1 2 2

Motif Candidate 3

Motif length: 12; Motif number: 16; Consensus: CCCCCCGCCACC

The profile matrix:

A 2 0 1 1 2 0 1 4 1 6 2 3

G 4 0 0 1 0 3 9 0 0 0 0 0

C 7 15 13 14 7 12 3 12 15 5 14 12

T 3 1 2 0 7 1 3 0 0 5 0 1

Motif Candidate 4

Motif length: 12; Motif number: 16; Consensus: CCCTTCCAGCCA

The profile matrix:

A 0 5 1 1 0 0 0 14 1 3 0 9

G 2 2 0 0 0 0 1 0 12 0 2 0

C 12 8 9 4 2 14 15 2 3 10 13 6

T 2 1 6 11 14 2 0 0 0 3 1 1

Motif Candidate 5

Motif length: 12; Motif number: 4; Consensus: GGGGGGGTGGGG

The profile matrix:

A 1 0 0 0 0 0 0 0 0 0 0 0

G 3 2 4 4 4 4 4 0 4 4 4 4

C 0 2 0 0 0 0 0 0 0 0 0 0

T 0 0 0 0 0 0 0 4 0 0 0 0

Motif Candidate 6

Motif length: 12; Motif number: 16; Consensus: CCTTTCCAGCCC

The profile matrix:

A 1 3 1 0 0 0 1 12 0 2 0 3

G 5 0 1 3 0 0 0 0 14 0 0 0

C 10 13 4 0 6 16 15 1 0 13 11 7

T 0 0 10 13 10 0 0 3 2 1 5 6

Motif Candidate 7

Motif length: 12; Motif number: 16; Consensus: ACCCTGCCAGCT

The profile matrix:

A 7 0 1 1 0 0 1 1 11 5 1 2

G 5 0 1 2 0 14 1 2 0 8 0 2

C 0 15 8 12 0 0 9 13 0 2 15 2

T 4 1 6 1 16 2 5 0 5 1 0 10

Motif Candidate 8

Motif length: 12; Motif number: 16; Consensus: CAGGAACCCCAG

The profile matrix:

A 4 11 0 7 9 8 5 1 3 0 8 1

G 1 1 16 9 7 0 0 5 1 0 0 11

C 10 0 0 0 0 7 8 10 12 16 0 4

T 1 4 0 0 0 1 3 0 0 0 8 0

Motif Candidate 9

Motif length: 12; Motif number: 6; Consensus: GCGGGGGTGGGG

The profile matrix:

A 1 0 0 2 0 0 0 0 0 2 0 1

G 4 2 5 4 6 6 3 0 6 4 6 4

C 0 3 0 0 0 0 3 0 0 0 0 0

T 1 1 1 0 0 0 0 6 0 0 0 1

Motif Candidate 10

Motif length: 12; Motif number: 2; Consensus: ATGAGTATTTAA

The profile matrix:

A 1 0 0 2 0 0 2 0 0 0 2 2

G 0 0 2 0 2 0 0 0 0 0 0 0

C 0 0 0 0 0 0 0 0 0 0 0 0

T 1 2 0 0 0 2 0 2 2 2 0 0

Motif Candidate 11

Motif length: 12; Motif number: 5; Consensus: GGGTGGGGCAGA

The profile matrix:

A 1 0 0 0 0 2 0 0 2 3 0 4

G 4 5 3 0 5 3 5 5 0 0 5 1

C 0 0 2 0 0 0 0 0 3 0 0 0

T 0 0 0 5 0 0 0 0 0 2 0 0

Motif Candidate 12

Motif length: 12; Motif number: 6; Consensus: GGGAGACTGAGG

The profile matrix:

A 0 0 0 2 1 2 0 0 0 6 0 1

G 6 5 6 2 4 2 2 0 6 0 6 4

C 0 0 0 1 0 1 4 0 0 0 0 1

T 0 1 0 1 1 1 0 6 0 0 0 0

Motif Candidate 13

Motif length: 12; Motif number: 6; Consensus: GGATGAGGCAGA

The profile matrix:

A 2 0 2 1 1 3 0 0 1 5 0 5

G 4 6 2 0 5 3 6 6 0 0 6 1

C 0 0 2 0 0 0 0 0 5 0 0 0

T 0 0 0 5 0 0 0 0 0 1 0 0

Motif Candidate 14

Motif length: 12; Motif number: 5; Consensus: AGGCAGGGAGAG

The profile matrix:

A 4 0 0 0 2 0 2 0 4 2 5 1

G 1 5 5 0 1 5 3 5 0 3 0 4

C 0 0 0 5 1 0 0 0 0 0 0 0

T 0 0 0 0 1 0 0 0 1 0 0 0

Motif Candidate 15

Motif length: 12; Motif number: 6; Consensus: AGGCAGAGAAAG

The profile matrix:

A 5 2 0 0 3 1 3 0 5 3 6 0

G 0 4 6 0 0 4 3 6 0 3 0 4

C 1 0 0 6 2 0 0 0 0 0 0 0

T 0 0 0 0 1 1 0 0 1 0 0 2

Motif Candidate 16

Motif length: 12; Motif number: 4; Consensus: AGGAAGAAGAAG

The profile matrix:

A 4 0 0 3 3 0 4 2 0 3 4 0

G 0 4 2 1 1 4 0 1 2 0 0 4

C 0 0 2 0 0 0 0 1 2 1 0 0

T 0 0 0 0 0 0 0 0 0 0 0 0

Motif Candidate 17

Motif length: 12; Motif number: 4; Consensus: AGGAGGACAGGA

The profile matrix:

A 4 0 0 4 0 0 3 1 3 0 1 2

G 0 3 4 0 4 4 0 0 0 3 3 1

C 0 0 0 0 0 0 0 3 0 1 0 1

T 0 1 0 0 0 0 1 0 1 0 0 0

Motif Candidate 18

Motif length: 12; Motif number: 4; Consensus: AGGACAGGAGGT

The profile matrix:

A 3 0 0 3 0 4 0 0 3 0 0 0

G 0 3 3 1 0 0 4 4 0 3 2 1

C 0 0 0 0 4 0 0 0 1 0 2 1

T 1 1 1 0 0 0 0 0 0 1 0 2

Motif Candidate 19

Motif length: 12; Motif number: 4; Consensus: GAGGACAGGAGG

The profile matrix:

A 0 4 0 0 2 0 3 0 0 2 0 0

G 4 0 4 3 0 0 1 4 4 1 2 2

C 0 0 0 0 2 4 0 0 0 1 0 2

T 0 0 0 1 0 0 0 0 0 0 2 0

Motif Candidate 20

Motif length: 12; Motif number: 4; Consensus: AGCAAGAAGAAG

The profile matrix:

A 4 0 0 3 2 0 4 2 0 3 4 0

G 0 4 1 1 2 4 0 1 2 0 0 4

C 0 0 3 0 0 0 0 1 2 1 0 0

T 0 0 0 0 0 0 0 0 0 0 0 0

Motif Candidate 21

Motif length: 12; Motif number: 4; Consensus: CCCAGAGACCCC

The profile matrix:

A 1 0 1 4 0 2 0 2 0 0 0 0

G 0 0 0 0 4 2 4 0 0 0 0 0

C 2 4 3 0 0 0 0 2 2 3 4 4

T 1 0 0 0 0 0 0 0 2 1 0 0

Motif Candidate 22

Motif length: 12; Motif number: 4; Consensus: CCTGCCCCCAAC

The profile matrix:

A 0 0 0 0 0 1 0 0 0 4 4 0

G 0 0 0 2 0 0 0 0 0 0 0 1

C 4 2 1 2 4 3 4 4 4 0 0 3

T 0 2 3 0 0 0 0 0 0 0 0 0

Motif Candidate 23

Motif length: 12; Motif number: 4; Consensus: TGGGGTCTCTGG

The profile matrix:

A 1 0 0 0 1 0 0 0 0 0 1 0

G 0 4 4 4 3 1 0 0 0 1 2 4

C 0 0 0 0 0 0 4 1 4 0 0 0

T 3 0 0 0 0 3 0 3 0 3 1 0

Motif Candidate 24

Motif length: 12; Motif number: 4; Consensus: AGGACAGGAGGG

The profile matrix:

A 4 0 0 2 0 4 1 0 2 0 1 1

G 0 4 4 2 1 0 3 4 1 4 3 3

C 0 0 0 0 3 0 0 0 0 0 0 0

T 0 0 0 0 0 0 0 0 1 0 0 0
